# Preoperative prediction of meningioma consistency using tumor-to-cerebellar peduncle intensity ratios on T2WI, DWI, and ADC maps

**DOI:** 10.1097/MD.0000000000047316

**Published:** 2026-01-16

**Authors:** Jung-Soo Park, Jong-Myong Lee

**Affiliations:** a Department of Neurosurgery, Chonbuk National University Hospital and Medical School, Chon-Ju, Republic of Korea.

**Keywords:** apparent diffusion coefficient, diffusion-weighted imaging, magnetic resonance imaging, meningioma, T2-weighted imaging, TCTI ratio, tumor consistency

## Abstract

Meningioma consistency is a critical factor in surgical planning, as firmer tumors are more difficult to resect and may increase operative time and risk. This study aimed to evaluate whether tumor-to-cerebellar peduncle T2-weighted imaging intensity (TCTI) ratios derived from T2-weighted imaging (T2WI), diffusion-weighted imaging (DWI), and apparent diffusion coefficient (ADC) maps can serve as reliable, noninvasive predictors of tumor consistency. This retrospective study included 102 patients with supratentorial WHO grade I meningiomas who underwent surgical resection between January 2021 and October 2024. Tumor consistency was classified intraoperatively as soft or hard. Preoperative 3T MRI data were analyzed to calculate TCTI ratios using signal intensities from T2WI, DWI, and ADC maps. Among the 102 tumors, 54 were classified as soft and 48 as hard. Soft tumors showed significantly higher signal intensities on T2WI and DWI, and higher ADC values. TCTI ratios from T2WI, DWI, and ADC were significantly correlated with consistency (*P* = .01, <.001, and <.001, respectively). Logistic regression analysis confirmed that TCTI ratios derived from T2WI (95% CI: 1.429–22.095; *P* = .013) and ADC values (95% CI: 0.008–0.477; *P* = .007) were independent predictors of tumor consistency. Receiver operating characteristic analysis identified optimal TCTI cutoffs of 1.42 for T2WI (sensitivity: 62.5%, specificity: 57.4%) and 1.14 for ADC (sensitivity: 60.5%, specificity: 81.4%). TCTI ratios derived from T2WI and ADC maps are effective, noninvasive markers for predicting meningioma consistency. Their use in preoperative imaging may enhance surgical planning and improve clinical outcomes.

## 1. Introduction

The majority of meningiomas are histologically benign.^[1,2]^ Surgical resection remains the primary treatment modality in most cases. However, gamma knife radiosurgery is often employed for tumors that are small or located in areas where complete resection is not feasible. Surgical complications are influenced by factors such as tumor size, the presence of an arachnoid plane, location, proximity to neurovascular structures, and tumor consistency.^[3]^

Among these, tumor consistency plays a critical role in planning the surgical approach and minimizing postoperative neurological deficits.^[4,5]^ Hard meningiomas are typically more challenging to resect and require longer operative times compared to softer ones.^[6]^ Therefore, preoperative prediction of tumor consistency is essential for optimal surgical planning.

Conventional magnetic resonance imaging (MRI), particularly T2-weighted imaging (T2WI), is often used preoperatively to estimate meningioma consistency.^[6]^ Some studies have suggested that soft meningiomas appear hyperintense on T2WI and hypointense on T1WI.^[7]^ However, other investigations have failed to demonstrate a reliable correlation between T2WI signal intensity and tumor consistency. While T1WI and T2WI allow for qualitative assessment of tumor signal intensity, they do not offer quantitative insight into tumor characteristics.^[8]^

Diffusion-weighted imaging (DWI) provides apparent diffusion coefficient (ADC) values, which reflect water molecule diffusion within tissues. Although DWI has been explored as a quantitative method to assess tumor consistency, its predictive value remains uncertain.^[9]^

Recent studies have proposed that higher water content in soft tumors correlates with hyperintensity on T2WI, whereas lower water content and increased collagen or calcification in hard tumors result in hypointensity.^[2,3,6,9-12]^ The cerebellar peduncle, which is unaffected by tumor compression, has been used as a reference to normalize T2WI signal intensity.^[13]^ The tumor-to-cerebellar peduncle T2-weighted imaging intensity (TCTI) ratio has been shown to correlate strongly with tumor consistency and offers a simple, reproducible method for standardization.^[2,6,11]^

This study aims to compare TCTI ratios derived from T2WI, DWI, and ADC maps with intraoperative tumor consistency, with the goal of identifying reliable preoperative imaging biomarkers for predicting meningioma consistency.

## 2. Materials and methods

This retrospective study was approved by the Institutional Review Board. Written informed consent was obtained from all participants.

### 2.1. Patients

We retrospectively reviewed 114 consecutive patients with supratentorial meningiomas who underwent craniotomy between January 2021 and October 2024. All patients had newly diagnosed, untreated meningiomas. Histopathological diagnosis was confirmed following surgical resection. Patients with World Health Organization (WHO) grade II or III meningiomas were excluded.

Tumor consistency was assessed intraoperatively and categorized as “soft” if the tumor could be removed using suction alone, and “hard” if removal required scissors or a Cavitron Ultrasonic Surgical Aspirator (CUSA; Valleylab) at high intensity. Specifically, tumors removed with a CUSA intensity < 70 were considered soft, whereas those requiring > 70 were considered hard. Tumors resected en bloc without the use of suction or CUSA were excluded.

We also excluded 7 patients with significant tumor calcification, 2 with intratumoral hemorrhage (due to MRI susceptibility artifacts), and 3 with small skull base tumors (<10 mm). Additional exclusion criteria included absence of 3.0 T MRI (due to metallic implants or devices), intratumoral hemorrhage on imaging, and failure to obtain informed consent.

After applying these criteria, 102 patients with 102 meningiomas were included. All surgeries were performed using the same CUSA device at our institution.

### 2.2. MR imaging

MRI examinations were conducted on a 3.0-T scanner (Achieva, Philips Medical Systems, Best, the Netherlands) using a 16-channel head coil. Imaging was performed in 3 orthogonal planes and included multiple conventional sequences. A non-contrast T1-weighted spin-echo sequence was obtained with the following parameters: repetition time (TR), 450 ms; echo time (TE), 18 ms; field of view (FOV), 22 cm × 22 cm; matrix size, 256 × 192; section thickness, 6 mm; interslice gap, 1.0 mm; and 2 signal averages.T2-weighted fast spin-echo images were acquired with TR = 4800 ms, TE = 100 ms, echo train length = 18, FOV = 22 × 22 cm, matrix = 512 × 320, section thickness = 6 mm, interslice gap = 1.0 mm, and one acquisition. For fluid-attenuated inversion recovery (FLAIR) imaging, the parameters were TR = 10,000 ms, TE = 140 ms, inversion time = 2400 ms, FOV = 22 × 22 cm, matrix = 288 × 160, section thickness = 6 mm, interslice gap = 1.0 mm, and 2 acquisitions. T2*-weighted gradient-echo images were obtained with TR = 600 ms, TE = 12 ms, FOV = 22 × 22 cm, matrix = 320 × 192, section thickness = 6 mm, interslice gap = 1.0 mm, and 1 acquisition. DWI was performed using *b*-values of 1000 and 4000 seconds/mm^2^ with an effective gradient strength of 40 mT/m and a slew rate of 150 mT/m/ms. The DWI protocol, acquired with an 8-channel phased-array head coil, used a TR of 5000 ms and TEs of 73.2 ms (*b* = 1000 seconds/mm²) and 100 ms (*b* = 4000 seconds/mm²). Additional parameters included 1 signal average, FOV = 22 × 22 cm, matrix = 128 × 128, section thickness = 6 mm, interslice gap = 1.0 mm, and 2 acquisitions, resulting in scan times of approximately 20 seconds and 40 seconds for each *b*-value, respectively.

ADC maps were generated using Functool software (GE Medical Systems) by calculating the signal intensity on diffusion-weighted images (DWI) at 2 different *b*-values (0 and 1000 seconds/mm^2^ for the ADC map with *b* = 1000, and 0 and 4000 seconds/mm^2^ for the ADC map with *b* = 4000) on a pixel-by-pixel basis.^[14]^

**Figure 1.**
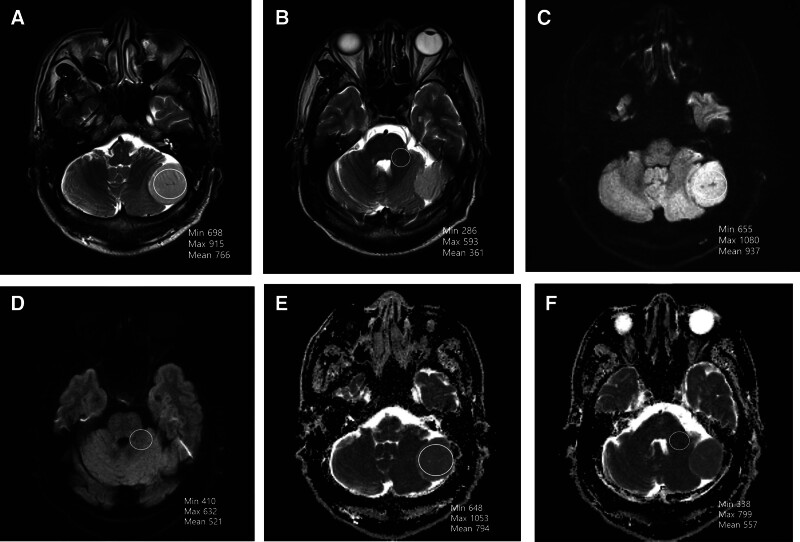
A 53-year-old woman with left cerebellar (soft meningioma). (A) Tumor ROI on T2-weighted MR sequence. (B) ROI in the middle cerebellar peduncle on T2-weighted MR sequence. The TCTI ratio = intensity value for ROI within tumor/intensity value for ROI within middle cerebellar peduncle** **= 698/286 = 2.44. (C) Tumor ROI on DWI MR sequence. (D) ROI in the middle cerebellar peduncle on DWI MR sequence. The TCTI ratio = intensity value for ROI within tumor/intensity value for ROI within middle cerebellar peduncle** **= 655/410 = 1.695. (E) Tumor ROI on ADC MR sequence. (F) ROI in the middle cerebellar peduncle on ADC MR sequence. The TCTI ratio = intensity value for ROI within tumor/intensity value for ROI within middle cerebellar peduncle** **= 648/338 = 1.670. ADC = apparent diffusion coefficient, DWI = diffusion-weighted imaging, ROI = region of interest, TCTI = tumor-to-cerebellar peduncle T2-weighted imaging intensity.

**Figure 2.**
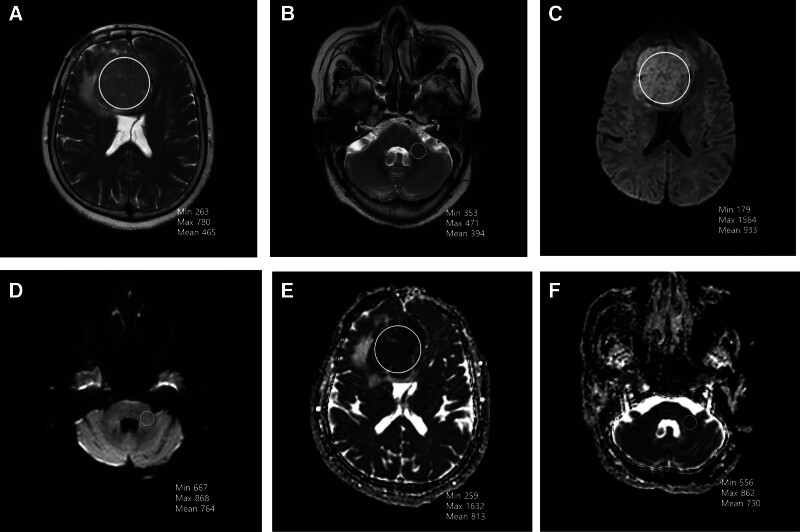
A 78-year-old woman with right frontal falx meningioma (hard meningioma). (A) Tumor ROI on T2-weighted MR sequence. (B) ROI in the middle cerebellar peduncle on T2-weighted MR sequence. The TCTI ratio = intensity value for ROI within tumor/intensity value for ROI within middle cerebellar peduncle** **= 263/353 = 0.745. (C) Tumor ROI on DWI MR sequence. (D) ROI in the middle cerebellar peduncle on DWI MR sequence. The TCTI ratio = intensity value for ROI within tumor/intensity value for ROI within middle cerebellar peduncle** **= 179/667 = 0.268. (E) Tumor ROI on ADC MR sequence. (F) ROI in the middle cerebellar peduncle on ADC MR sequence. The TCTI ratio = intensity value for ROI within tumor/intensity value for ROI within middle cerebellar peduncle** **= 259/556 = 0.465. ADC = apparent diffusion coefficient, DWI = diffusion-weighted imaging, ROI = region of interest, TCTI = tumor-to-cerebellar peduncle T2-weighted imaging intensity.

TCTI ratios were calculated using T2-weighted sequences as described by Smith et al^[6]^ (Figs. 1A, B and 2A, B). Regions of interest (ROIs) were manually drawn within tumors to obtain average signal intensity. For homogeneous tumors, a single large ROI was used; for heterogeneous tumors, multiple ROIs were averaged to derive a representative value. The middle cerebellar peduncle was selected as the internal reference region due to its stability across patients and insensitivity to tumor-induced compression. This normalization addressed inter-scanner variability in signal intensity.^[15]^

ROIs were drawn on all slices where the tumor was visible, and minimum (ADCmin), maximum (ADCmax), and mean (ADCmean) values were recorded. Blinded to patient information, 2 radiologists with more than 5 years of experience in image reading manually segment region of interest (ROI) for all data, including the largest cross-sectional area of the tumor ROI. A senior neuroradiologist with over twenty years of experience in image reading reviewed and selected ROIs used to radiomic features extraction.

### 2.3. Statistical analysis

All statistical analyses were conducted using SPSS version 14.0 (SPSS, Chicago). Descriptive statistics were used to summarize patient demographics. The association between imaging features and intraoperative tumor consistency was evaluated using the Mann–Whitney test. Odds ratios (ORs) with 95% confidence intervals (CIs) were used to assess the strength of associations.

Multivariate logistic regression was performed to determine the predictive value of imaging variables for tumor consistency. Receiver operating characteristic curve analysis was used to identify optimal cutoff values for significant predictors, with sensitivity, specificity, and predictive values reported. A *P*-value < .05 was considered statistically significant.

## 3. Results

A total of 102 patients with supratentorial meningiomas were included in the final analysis. The cohort consisted of 26 men and 76 women, with a mean age of 58.1 years (range: 19–85 years). Among these, 54 tumors were classified intraoperatively as soft and 48 as hard based on surgical handling characteristics (Table 1).

**Table 1:** Baseline characteristics.

	Soft meningioma (N = 54)	Hard meningioma (N = 48)	*P*-value
Age (mean)	60.04	55.94	.084
Sex (male/female)	(9/45)	(17/21)	.031
Tumor T2WImin	608.18	403.64	.009
Tumor T2WImax	1630.0	1083.3	.14
Tumor T2WImean	953.8	767.45	.036
TCTIof T2WI	1.34	1.62	.001
Tumor DWImin	652.05	530.05	.011
Tumor TDWImax	902.85	769.33	.022
Tumor DWImean	720.95	817.48	.035
TCTIof DWI	0.88	1.26	<.001
Tumor ADCmin	464.20	434.68	.012
Tumor ADCmax	764.14	855.64	.024
Tumor ADCmean	817.47	772.03	.019
TCTI of ADC	1.37	1.16	<.001

ADC = apparent diffusion coefficient, DWI = diffusion-weighted imaging, T2WI = T2-weighted imaging, TCTI = tumor-to-cerebellar peduncle T2-weighted imaging intensity.

There was no significant relationship between patient age and tumor consistency. However, sex was found to be significantly associated with tumor texture, with hard meningiomas occurring more frequently in male patients (*P* = .03).

Radiological evaluation revealed meaningful associations between tumor consistency and imaging characteristics. On T2WI, soft tumors tended to exhibit higher signal intensities. Although T2WI minimum and maximum intensities showed a borderline association (*P* = .09 and *P* = .14), T2WI mean intensity was significantly greater in the soft group (*P* = .036, respectively). Similarly, DWI revealed that soft tumors had higher minimum, maximum, and mean signal intensities, with statistically significant differences across all measures (*P*-values ranging from .011 to .035; Figs. 1C, D and 2C, D).

ADC analysis also revealed significant differences between soft and hard tumors. The soft group demonstrated higher minimum (*P* = .012), maximum (*P* = .024), and mean (*P* = .019) ADC values, supporting the hypothesis that softer tumors allow greater water diffusion (Figs. 1E, F and 2E, F).

Normalization of signal intensity using the TCTI ratio provided further insight. TCTI ratios from T2WI, DWI, and ADC maps were all significantly associated with tumor consistency (*P* = .01, <.001, and <.001, respectively). Specifically, the mean TCTI ratio from T2WI was 1.34 ± 0.45 in the soft tumor group and 1.62 ± 0.28 in the hard tumor group (*P* = .003). For DWI, the mean TCTI ratio was 0.88 ± 0.23 in the soft group and 1.26 ± 1.07 in the hard group (*P* = .025). In contrast, the mean TCTI ratio from ADC values was higher in the soft group (1.37 ± 0.10) compared to the hard group (1.16 ± 0.18), with a highly significant difference (*P* < .001).

Logistic regression analysis confirmed that TCTI ratios derived from T2WI (95% CI: 1.429–22.095; *P* = .013) and ADC values (95% CI: 0.008–0.477; *P* < .007) were independent predictors of tumor consistency (Table 2). In contrast, TCTI from DWI and minimum T2WI signal intensity did not reach statistical significance in the adjusted model (*P* = .127 and *P* = .146).

**Table 2:** Logistic regression analysis.

	Soft group (N = 54)	Hard group (N = 48)	*P*-value	Odds ratio	95% CI
Sex (M/F)	9/45	17/21	.201	2.150	0.665–6.945
Tumor T2 min	608.18	403.64	.146	0.999	0.997–1.000
T2 TCTI	1.34	1.62	.013	5.615	1.429–22.095
DWI TCTI	0.88	1.26	.127	3.616	0.693–18.872
ADC TCTI	1.37	1.16	.007	0.064	0.008–0.477

ADC = apparent diffusion coefficient, DWI = diffusion-weighted imaging, TCTI = tumor-to-cerebellar peduncle T2-weighted imaging intensity.

Receiver operating characteristic curve analysis further validated these findings. The optimal TCTI cutoff on T2WI was 1.42 (*P* = .001, 95% CI: 0.587–0.790), and with that cutoff point, the sensitivity, specificity, positive predictive value (PPV), and negative predictive value were 62.5%, 57.4%, 56.6%, and 63.2%, respectively. For ADC-derived TCTI, a cutoff of 1.14 offered strong performance (*P* < .001, 95% CI 0.181–0.385). With that cutoff point, the sensitivity, specificity, positive predictive value, and negative predictive value were 60.5%, 81.4%, 69.1%, and 84.4%.

## 4. Discussion

In this study, we demonstrated that TCTI derived from T2WI and ADC maps are significantly associated with meningioma consistency. Preoperative knowledge of tumor consistency is valuable for surgical planning, including operative time estimation and patient counseling.^[16]^

Accurately predicting tumor consistency preoperatively would serve as a clinically useful biomarker to guide neurosurgical strategies. Although some investigators have proposed that histological features – particularly fibrous content – contribute to tumor hardness,^[8,17]^ other studies have failed to demonstrate a strong correlation between histology and intraoperative consistency.^[18]^ Smaller case series have suggested that lower T2 signal intensity may be indicative of increased collagen content, with fibrous tumors often appearing isointense relative to cerebral tissue.^[19,20]^

There is a general consensus that decreased T2 signal intensity corresponds to firmer meningiomas.^[20]^ This finding is consistent with the hypothesis that T2 hypointensity reflects a higher proportion of fibrous or calcified tissue, whereas T2 hyperintensity indicates greater water content.^[21]^ Tumor cellularity and extracellular matrix composition, particularly the degree of fibrous tissue, influence signal intensity. Dense cellular regions with reduced extracellular space and a low nucleus-to-cytoplasm ratio typically exhibit low T2 signal intensity, while abundant extracellular water increases signal intensity.^[12,17,20]^

However, multiple factors can affect T2 signal intensity, which may limit its standalone predictive value for consistency.^[9,20,22,23]^ Previous studies have proposed that ADC values are inversely related to tumor cellularity and fibrous tissue content, suggesting that meningiomas with low ADC values are likely to be firmer.^[23-25]^

Our findings confirm that T2WI hypointensity is a reliable indicator of hard consistency, aligning with prior studies.^[2,3,6,9,11,16]^ Moreover, we demonstrated that TCTI ratios calculated from T2WI, DWI, and ADC values provide meaningful predictive value. This supports the approach proposed by Rabiee et al,^[13]^ who reported a correlation between higher TCTI values and softer tumors. The present study builds on prior work by showing that TCTI ratios from multiple imaging modalities can serve as robust, quantifiable predictors of tumor consistency.

Smith et al previously showed that all firm meningiomas had a TCTI ratio < 1.41.^[2]^ Consistently, we found that most hard tumors had TCTI ratios below 1.42, with significant differences between the soft and hard groups.

ADC values have been proposed as surrogates for tumor stiffness.^[23,26]^ Tumor cellularity and fibrous tissue contribute to the consistency of tumors, and it is proposed that the minimum ADC value is significantly correlated with meningioma consistency.^[10]^ It has been suggested that the ADC value inversely correlates with tumor cellularity and the amount of fibrous tissue within heterogeneous tumors.^[23–25,27,28]^ Although tumor cellularity and fibrous tissue content influence consistency, their contribution to ADC values varies. Miyoshi et al reported that combining standard and shifted ADC maps can enhance predictive accuracy for meningioma consistency.^[29]^

In our study, maximum ADC values were significantly lower in hard meningiomas compared to soft ones. However, there were no statistically significant differences in minimum or mean ADC values between the 2 groups. This contrasts with the findings of Yogi et al, who reported that minimum ADC values were significantly lower in hard meningiomas.^[10]^ These discrepancies may stem from differences in ROI placement or tumor heterogeneity. Notably, our study is the first to report on the use of ADC-based TCTI ratios as a consistency predictor. We found that a TCTI cutoff of 1.175 for ADC yielded a sensitivity of 82.0% and specificity of 55.8. Although ADC values have been widely proposed as predictors of tumor consistency, our findings revealed some inconsistencies. Specifically, while maximum ADC values were significantly lower in hard meningiomas, minimum and mean ADC values did not show statistically significant differences between soft and hard groups. This discrepancy contrasts with prior studies reporting strong correlations between minimum ADC values and tumor stiffness.^[10,25]^

Several limitations should be acknowledged. First, this was a retrospective, single-center study, which may introduce selection bias. Second, ROI placement was performed manually and is subject to observer variability. Third, intraoperative assessment of consistency remains subjective and may vary between surgeons.

## 5. Conclusion

This study demonstrates that meningioma consistency is significantly associated with MRI signal characteristics, particularly TCTI ratios derived from T2WI, DWI, and ADC maps. Among these, TCTI ratios from T2WI and ADC were identified as independent predictors of tumor consistency in multivariate analysis.

These findings suggest that preoperative MRI-based TCTI assessment may serve as a reliable, noninvasive biomarker to predict tumor texture. Incorporating this information into surgical planning could improve operative efficiency, assist in selecting appropriate surgical tools, and enhance patient counseling.

Further prospective, multicenter studies are warranted to validate the clinical utility of TCTI ratios and establish standardized imaging protocols for routine preoperative evaluation of meningioma consistency.

## Author contributions

**Conceptualization:** Jong-Myong Lee.

**Methodology:** Jung-Soo Park.

**Supervision:** Jong-Myong Lee.

**Writing – original draft:** Jung-Soo Park.

**Writing – review & editing:** Jong-Myong Lee.
